# Low frequency of HPV positivity in breast tumors among patients from south-central Poland

**DOI:** 10.1186/s13027-021-00405-z

**Published:** 2021-11-27

**Authors:** Beata Biesaga, Anna Janecka-Widła, Marta Kołodziej-Rzepa, Anna Mucha-Małecka, Dorota Słonina, Marek Ziobro, Joanna Wysocka, Agnieszka Adamczyk, Kaja Majchrzyk, Joanna Niemiec, Aleksandra Ambicka, Aleksandra Grela-Wojewoda, Agnieszka Harazin-Lechowska

**Affiliations:** 1 Center for Translational Research and Molecular Biology of Cancer, Maria Sklodowska-Curie National Research Institute of Oncology, Gliwice Branch, Wybrzeże Armii Krajowej 15, 44-101 Gliwice, Poland; 2 Department of Tumor Pathology, Maria Sklodowska-Curie National Research Institute of Oncology, Cracow Branch, Gliwice, Poland; 3 Department of General, Oncological and Vascular Surgery, 5th Military Clinical Hospital, Cracow, Poland; 4 Department of Radiotherapy, Maria Sklodowska-Curie National Research Institute of Oncology, Cracow Branch, Gliwice, Poland; 5 Department of Clinical Oncology, Maria Sklodowska-Curie National Research Institute of Oncology, Cracow Branch, Gliwice, Poland; 6 Institute of Medical Sciences, Medical College of Rzeszow University, Rzeszow, Poland; 7 Department Laboratory of Medical Diagnostics, Cytogenetics and Flow Cytometry Specialist Hospital, Brzozow, Poland

**Keywords:** Breast cancers, HPV infection, South-central Poland

## Abstract

**Background:**

Some studies suggest that Human Papilloma Virus (HPV) infection is important factor in carcinogenesis of breast tumors. This study’ objective was to analyze HPV prevalence in breast cancers of patients from south-central Poland.

**Materials and methods:**

The study was performed based on archival paraffin embebbed and formalin fixed blocks in the group of 383 patients with breast cancer. HPV prevalence and its genotype were assessed, respectively by: nested PCR (with two groups of primers: PGMY09/PGMY11 and GP5+/GP6+), quantitative PCR (qPCR). Tumors were classified as HPV positive in case of at least one positive result in nested PCR and positive results in genotyping procedure. For all HPV positive tissues P16 immunostaining was applied in order to confirm active viral infection.

**Results:**

In the group of 383 breast cancers, HPV positivity was found in 17 samples (4.4%) in nested PCR. All these samples were subjected to HPV genotyping. This analysis revealed presence of HPV type 16 into two tumors (0.5%). In these two cancers, P16 overexpression was reported.

**Conclusion:**

In breast tumors of patients from south-central Poland in Poland, HPV positivity is demonstrated in very low percentage of cases.

## Introduction

According to the analysis based on the GLOBOCAN database from 2018, 2.2 million infection-attributable cancers (13% of all cancer cases) were diagnosed [1]. The four most important pathogens related with increase of cancer risk were: *Helicobacter pylori*, high risk *Human Papillomavirus* (HR-HPV) and *Hepatitis B* and C viruses. In 2018, HR-HPV infection was responsible for 690 000 new diagnosed cancers [1]. This infection (mainly HPV16), is an approved risk factor in development of some anogenital cancers (cervical, vulvar, vaginal, penile and anal cancers) and tumors localized in head and neck region. At present, HR-HPV infection is also proposed as a risk factor in development of breast cancers (BC). In a few meta-analyses [2–6], it was shown that exposure to HR-HPV is related to increase of relative risk of breast cancer development from 3.24 [4] to 5.9 [5].

HPV presence in breast cancers can occur from direct skin to skin contact during sexual intercourse or in case of woman with previous history of HPV positivity in the cervix trough nipple or micro-lesions or body fluids (bloods, lympha) [7]. It is hypothesis that this infection may be involved in the early stage of BC carcinogenesis. It has been shown that viral presence is related with overexpression of inflammatory cytokines [8, 9] and of cyclooxyganse-2 and cytidine deaminase (APOBEC3B), which causes significant γH2AX focus formation or DNA breaks [10].

Data concerning prevalence of HPV in breast cancers are, however, inconsistent. The percentage of HPV positivity in these type of tumors varied between individual studies from 0.0 [4, 11–21] to 86.2% [22]. Some of meta-analyses revealed geographical differentiation of HPV infection in BC, showing the higher percentage of infection in middle-east Asia and in both Americas [2] or in Oceania and Asia [6] and the lowest in Europe [2, 6]. However, data concerning HPV prevalence in BC from Europe are also inconsistent, with the percentage of HPV positivity ranged from 0% [11, 12, 17, 18, 20, 21] to 64% [23]. It is also worth to noticed that all European studies concerning HPV presence in breast cancer, except one, came from Western Europe. One exception is the Polish study from 2013 [24], in which HPV prevalence was analysed in 60 FFPE breast cancer tissues and viral presence was found in 8 samples (13.3%). It should be also mentioned that in all above-mentioned European researches the number of analysed BC tissues varied from 11 [23] to 251 [25] samples, with median value at the level of 74. Taking all these facts into account, the aim of the present study was to analyse HPV prevalence in 383 formalin fixed and paraffin embedded and formalin FFPE tissues of breast cancers—according to our best knowledge for the first in Europe in so large group of invasive ductal breast cancer samples. HPV prevalence and its genotype as well as active HPV infection were assessed, respectively by: nested PCR (with two groups of primers: PGMY09/PGMY11 and GP5+/GP6+), quantitative PCR (qPCR) and P16 immunostaining.

## Materials and methods

### Study population

Initially, formalin–fixed paraffin emedded tissue blocks (FFPE) were gathered from 448 patients with infiltrating invasive ductal breast cancer in clinical stage T1–2, N1–2, M0, treated in Maria Skłodowska-Curie Memorial Cancer Centre and Institute of Oncology, Krakow Branch, Poland between 1992 – 2006 (primary material analyzed in grants: N401 173 31/3808, NN 401 096 137, NN401 2344 33 financed by the Polish Ministry of Science and Higher Education, and DEC-2013/09/B/NZ5/00764 financed by the Polish National Science Centre).

The study was approved by the Ethical Committee at the Regional Medical Chamber in Cracow (Poland) (No. 11KBL/OIL/2009 and 12KBL/OIL/2009, in the case of DEC-2013/09/B/NZ5/00764, decision of 4 December 2013). Informed consent was obtained from all individual participants included in the study. All samples were anonymized.

### Study design

For all cancers, their immnunophenotypes was assessed based on estrogen, progesterone, and HER2 immunoexpression (or hybridization in situ in case of inconclusive results of HER2 immunostaining), according to St. Gallen International Expert Consensus on The Primary Therapy of Early Breast Cancer 2013 [26]. Before DNA extraction, each paraffin block underwent histopathological verification in order to confirm diagnosis (tumor type, grade) and indicate paraffin blocks with biopsy or surgical material with at least 50% of tumor neoplasm for DNA extraction. Due to low amount of cancer tissue in paraffin blocks, DNA extraction was possible for 383 patients. Detailed characteristics of these 383 patients is present in Table 1. Briefly, women aged from 27 to 84 years (with mean and median values 53.5 years ± 0.59 and 53.5 years, respectively), 54.7% have tumors in clinical stage T1N1, 23.3% in stage T1N2, 18.0% in T2N1 and 4.0% in T2N2. Among 383 tumors predominate those with luminal B HER2^+^ (32.6%) and luminal B HER2^−^ (24.1%) immunophenotypes.

**Table 1:** Clinical characteristic of patients with breast cancer involved in the study

Characyeristics	Numeber of cases	%
All (%)	383	100.0
*Age*		
< 50 years	124	32.4
≥ 50 years	259	67.6
*Tumour size*		
T1	108	28.2
T2	268	70.0
T3	7	1.8
*Nodal status*		
N0	53	13.8
N1	238	62.2
N2	79	20.6
N3	13	3.4
*Grade*		
G1	37	9.7
G2	145	37.8
G3	201	52.5
*Oestrogen receptor status*		
Positive	280	73.1
Negative	103	26.9
*Progesterone receptor status*		
Positive	263	68.7
Negative	120	31.3
*HER2 status*		
Overexpressing	187	48.8
Not overexpressing	196	51.2
*Ki-67LI*^*a*^		
≤ 19.7%	101	26.4
> 19.7%	282	73.6
*Breast cancer immunophenotypes*^*b*^		
LA	56	14.6
LB HER2^−^	92	24.1
LB HER2^+^	125	32.6
HER2^+^	59	15.4
TN	51	13.3
*HPV infection—nested PCR*		
Yes	17	4.4
No	366	95.6
*HPV infection—qPCR*		
Yes	2	0.5
No	381	99.5
*P16 immunoespression*		
Yes	2	0.5
No	381	99.5

Ki-67LI Ki-67 labelling index, LA luminal A, LB luminal B, TN triple negative

^a^Cut-off point from minimal *P* value method

^b^Immunophenotypes indicated on the basis of ER, PgR, HER2 and Ki-67 expression according to St. Gallen International Expert Consensus on The Primary Therapy of Early Breast Cancer 2013[26]

### DNA extraction

DNA was extracted using 4 μm thick FFPE Sects. (3–7 depending on sample size) using ReliaPrep™FFPE gDNA Miniprep System (Promega, Madison, USA) based on manufacturer`s suggestions with our own modification. All details concerning this procedure were described earlier [27]. In brief, after 1 min incubation with mineral oil at 80 °C, addition of Solution Buffer and centrifugation, samples were incubated with Proteinase K for the whole night at 56 °C (own modification), and for 1 h at 80 °C. After cooling, RNAse A treatment and incubation with mixture of BL Buffer and 100% ethanol, the aqueous phase was transferred to the Binding Column DNA. DNA was eluted with 50 μl of Elution Buffer. Quantity and quality (A260/280 and A260/230 ratios) of extracted DNA were assessed spectrophotometrically with Biophotometer Plus (Eppendorf AG, Hamburg, Germany). DNA samples were stored at − 20 °C until analysed. In order to check pattern of DNA degradation, each sample was subjected to qPCR for amplification of 139 bp fragment of *β-actin* gene using TaqMan® Gene Expression Assay (Thermo Fisher Scientific, Waltham, USA), with mix of specific primers and MGB probe as described earlier [27].

### Nested PCR

**Fig. 1:**
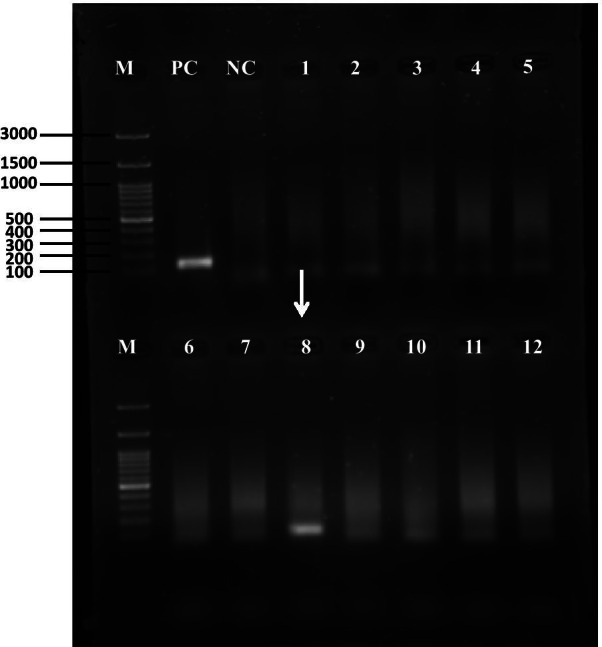
Gel picture of amplicons obtained after nested polymerase chain reaction with two pairs of primers—outer (PGMY09/PGMY11) and inner (GP5+/GP6+, both Genomed) for the detection of human papilloma virus. Both PCR were carried out using 3 μl of the DNA template and amplicons, the final products were separated electrophoretically in 2% agarose gel and visualized using SimplySafe dye (EURx, Poland). Line 1: molecular marker, line PC: positive control (DNA from HPV16-positive squamous cell carcinoma of cervix extracted from cancer tissue obtained from biopsy before cancer treatment, size of the band—150 bp, line NC: negative control (water instead of DNA), lines 1–12: samples of breast cancer. Arrow indicates HPV-positive sample of breast cancer (sample code 561,482, size of the band—150 bp). The light bands in negative lanes most likely come from highly degraded DNA extracted from FFPE tissues. Genomic DNA extracted from FFPE tissues is highly degraded due to the cross-linking between nucleic acid strands and proteins, as well as random breakings in sequence

The nested PCR involves two pairs of primers—outer (PGMY09/PGMY11and inner (GP5+/GP6+, both Genomed), used in two successive PCR runs, what allows to detect L1 gene fragment of multiple HPV types during one experiment. The product of the first reaction serves as a template in the second reaction. Sequences of primers, composition of reactions mix and conditions of PCR reactions were described earlier [28]. To assess the specificity of the nested PCR with PGMY09/011 and GP5+/6+ primers, DNA from HPV16-positive squamous cell carcinoma of cervix extracted from cancer tissue (FIGO stage IIB) obtained from biopsy before cancer treatment was analyzed performed using. The DNA sample with HPV16 positivity was identified based on TaqMan-based 5’exonuclease quantitative PCR with type-specific primers in our earlier study [29]. In each run of nested PCR water as negative control and DNA from HPV16-positive squamous cell carcinoma of cervix (FIGO stage IIB) extracted from cancer tissue obtained from biopsy before cancer treatment as positive control were used. The final products were separated electrophoretically in 2% agarose gel and visualized using SimplySafe dye (EURx, Poland) (Fig. 1). For each tumor 2 analyses were performed.

### HPV genotyping

For all HPV positive samples in nested PCR (at least one positive result), virus genotyping with AmoyDx® Human papillomavirus (HPV) Genotyping Detection Kit (Amoy Diagnostics Co., LTD, China) was performed. This procedure allows for genotyping of 19 high risk HPV (16, 18, 26, 31, 33, 35, 39, 45, 51, 52, 53, 56, 58, 59, 66, 68, 70, 73 and 82) and 2 low risk (6 and 11) on the basis of virus *L1* gene amplification. The reaction was carried out according to manufacturer`s protocol and all its details were presented earlier [28]. As a negative control, to each experiments water instead template was added. HPV genotype was determined by analysis of combination of fluorescent signals from FAM, CY5 and HEX/VIC in each tube according to manufacturer’s instruction. Tumors were classified as HPV positive in case of at least one positive result in nested PCR and positive results in genotyping procedure [28].

### P16 immunostaining

**Fig. 2:**
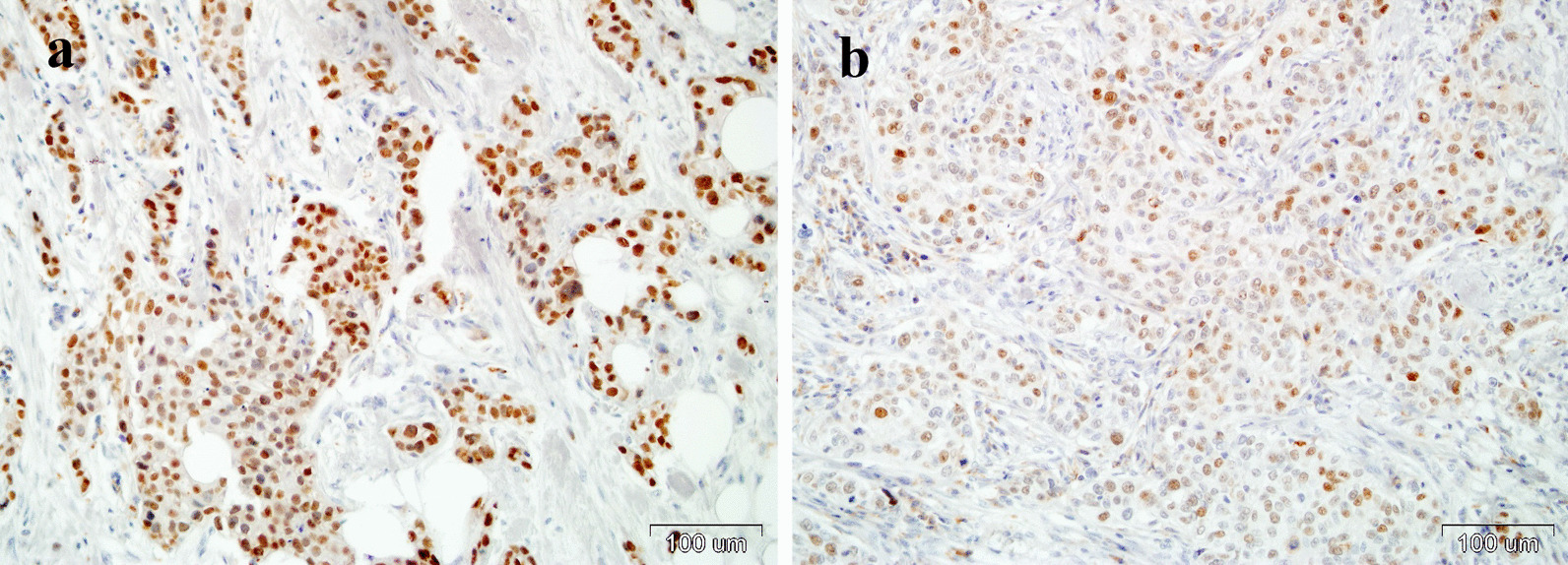
Immunohistochemical staining of P16 using CINtec® Histology Kit (Roche, Heidelberg, Germany) in formalin-fixed paraffin-embedded samples from two breast cancers with HPV positivity: **a** sample code 283,618, **b** sample code 561,482. Magnification ×200

Expression of P16 was analyzed in the group of 383 tumors using CINtec® Histology Kit (Roche, Heidelberg, Germany) according to the manufacturer’s procedure, described by us earlier [28]. In brief, 4 µm thick sections of FFPE HNSCC tissues were deparaffinized and hydrated through a series of xylenes and alcohols. After antigen unmasking (96 °C, 10 min) and exogenous peroxidases quenching (5 min), sections were incubated with primary anti-p16 antibody (clone E6H4, RT, 30 min) followed by 30 min incubation with visualization system. P16 was visualized using DAB (3, 3′–diaminobenzidine) and for nuclear counterstaining haematoxylin was applied. Cervical cancer tissue with p16 overexpression was used as a positive control. For negative control, the primary antibody was omitted. Immunopositivity was defined according to Lewis et al. [30] as follows: > 75% of positive staining cells or > 50% staining with > 25% confluent areas of positive staining (Fig. 2).

## Results

### Nested PCR

For all 383 DNA samples (100.0%) extracted from FFPE, amplification of β-action was noticed. HPV positivity in nested PCR was found in 17 samples (4.4%), at least in one reaction in 15 samples and in two reactions in two cases (Fig. 1).

### HPV genotyping and P16 immunostaining

All these samples were subjected to HPV genotyping. This analysis revealed HPV presence in two tumors coded as: 283,618, and 561,482 (Table 2). In case sample codded as 506,594, the curve of amplification start to increase, however it did not achieve the threshold. For the remaining 14 tumors, the curves of amplification did not increase during genotyping procedure. Because we assumed that HPV positivity is recognized when at least one positive result in nested PCR and positivity in genotyping procedure were found, we state that among 383 samples, HPV infection was noticed in 2 tumors (0.5%). Two HPV-positive breast carcinoma cases were infiltrating ductal carcinoma, not otherwise specified types (according to the newest WHO 2019 classification, ICD-O code 8500/3) with high histological grade (G3), almost no tubule formation, areas of necrosis, high mitotic index—over 20/10 *high* power fields and marked nuclear pleomorphism (Table 2). Both of these cancers were infected with HPV16 and are characterized by P16 overexpression (Fig. 2), what indicate active viral infection.

**Table 2:** Characteristics of patients with HPV positive breast cancers in PCR nested and qPCR genotyping (Amoy test)

Feature	Sample code	
	283,618	561,482
*Clinical and histopathological features*		
Age	46	56
Clinical stage	T2N1	T2N2
Immunophenotype	Lum B HER2^+^	Lum B HER2^+^
Microscopic characteristics	tightly packed nests of neoplastic cells, with central necrosis, fibrosis and calcification	loosely packed nests of neoplastic cells with areas of necrosis
Grade	3 tubule formation 3 points + nuclear pleomorphism 3 points + mitotic count 3 points	3 tubule formation 3 points + nuclear pleomorphism 3 Points + mitotic count 2 points
Tumour margin	Infiltrative	Infiltrative
Tumour infiltrating lymphocytes	< 5%	About 10%
Lympho-vascular involvement	Absent	Absent
*DNA extraction*		
Total DNA concentration ± SE [µg]	5.43 ± 0.61	4.30 ± 0.32
A260/280	1.74 ± 0.10	1.91 ± 0.11
A260/230	1.94 ± 0.10	1.99 ± 0.14
Amplification of β-actin Ct ± SE	29.8 ± 0.4	29.2 ± 0.1
Nested PCR	++	++
HPV genotyping by qPCR	HPV16	HPV16
P16 immunostaining	Positive	Positive

*PCR* quantitative polymerase chain reaction

## Discussion

In the present study, in the group of 383 patients with invasive ductal breast cancers, (according to our best knowledge the largest studied according to HPV incidence in Poland and Europe and one of the largest worldwide), we have found HPV prevalence at the level of 0.5% (2 cases). These two tumors were also characterized by P16 overexpression (Fig. 1), what indicates active viral infection [28]. Similar to us, some other authors from Italy [31], Iran [32] and Thailand [33] have shown low percentage (in range 3.6–5.5%) of HPV positivity among BC. In turn, in reports from Denmark [11], Greece [12], Spain [17], France [19], Swiss [20], United Kingdom [21] and Iran [13–15], China [4, 16] and Mexico [18] no HPV presence in BC was noticed. Based on results of current study and all above-mentioned reports, it can be concluded that HPV infection has no significance in the development of BC. However, there are many other studies, in which the percentage of HPV positivity in BC was much higher, in the range from 7.5 [34]–86.2% [22]. Moreover, in a few meta-analyses, HPV positivity in BC was noticed in 23.0 [5]–30.3% [4] of BC. These meta-analyses reported also that HPV infection causes increase in summary odds ratio concerning risk of BC development from 3.24 [4] to 5.9 [5]. Analysing all reports concerning HPV prevalence in BC, it can be distinguish several factors affecting these contradictionary results, such as geographical region, characteristics of patient’s group and HPV detection methods.

In relation to the hypothesis about geographical differenattion of HPV presence in breast cancer, it should be noticed that it is in contradiction to the results of our study and other study from from south-west Poland [24], in which the prevalence of HPV infection was found at the higher level (13.3%) as compared to us. Similarly, contrary results came from other countries, such as: Greece (range 0 [12]–15.9% [35]) Italy (range 3.6 [31]–25.5% [36]), Spain (range 0 [17]–51.8% [25]) or United Kingdom (range 0 [21]–47.0% [37]). Therefore, it seems that other factors influence heterogeneity in HPV prevalence in BC between individual studies. One of these factors may be heterogeneity of patient’s group according to such clinical and histopathological features, as patient’s age and BC histological type.

Considering the influence of BC histological type on HPV prevalence, it should be noticed that some authors [10, 24] have found higher frequency of HPV positivity in invasive lobular carcinomas (50% [24] and 100% [10]) than in invasive ductal carcinomas (8.7% [24] and 30.0% [10]), whereas in the current study all tumors were invasive ductal (Table 2). In turn, Piana et al. [34], analysing 80 BC, most of which were ductal, have found, similar to us, HPV positivity at relative low level (7.5%). However, in many studies reverse correlation, i.e. higher proportion of HPV positivity in ductal than in lobular breast cancers [25] or no correlation between these two parameters were found [38–40]. On the other hand, considering histological type of BC, attention must be pay on such factors as grade and BC immunephenotypes. In the current study, two HPV positive tumors were in grade III (Table 2). This finding is in agreement with results of Kroupis et al. [35], who have demonstrated that 70.6% of HPV positive BC were in grade III, whereas in HPV negative BC it was 33.3%. These results are also indirect confirmed by some reports showing significant higher percentage of HPV presence in faster proliferating cancers compared to tumors characterized by lower proliferation [25, 41]. Some authors have also suggested the relation between HPV infection and BC immunophenotypes. In our study, all BC with HPV infection were HER2 positive. Similar to us, Carolis et al. [41] have shown significantly higher percentage of HPV infection among HER2 overexpressed and triple negative BC comparing to oestrogen or progesterone positive cancers. In turn, Piana et al. [34] and Corbex et al. [42] have obtained the significantly higher percentage of HPV positivity in TNBC as compared to non-TNBC. All these findings suggest that HPV presence is related with higher aggressiveness of tumors, although it should be taken into account that some other authors have obtained contrary results, i.e. significantly higher percentage of HPV infection in tumors overexpressed oestrogen and/or progesterone receptors [10, 43] or lack of correlation between HPV infection and BC grade or immunophenotypes [25, 38, 40].

The other factors influencing on contr dictionary results concerning HPV incidence in BC are related with methods using to assess HPV positivity. Considering the influence of PCR based techniques (mostly applied to assess viral presence in breast cancers), special attention must be paid on type of material using as a source of HPV DNA. In the present study, we have used nested PCR with GP5+/6+, MY09/11 and PGMY09/11 set. In relation to this set, we would also like to pay attention on results of Erhart et al. [44], who have compared GP5+/6+, MY09/11 set and PGMY09/11 primers set for the detection of viral DNA by single step PCR and nested PCR in FFPE tissues from oral squamous cell carcinomas. These authors have found that single step PCR with GP5+/6+ and MY09/11 primers and MY/GP+ nested PCR did not amplify HPV DNA in any samples. PGMY09/11 primers detected HPV DNA in 13.0% of OSCC cases and this rate was raise to 17.4% with the use of PGMY/GP+ nested PCR (the same combination of primers as in our study). They concluded that the PGMY/GP+ nested PCR is the most appropriate primer set for the detection of HPV DNA using FFPE samples from OSCC. In turn, Božić et al. [45] have compared HPV detection rate in FFPE of head and neck carcinoma using three amplification methods: single PCR and real-time PCR and nested PCR. In their study there was not HPV amplification in any of the 50 FFPE samples using the single PCR and real‐time PCR, whereas HPV DNA was detected in 22% of samples using nested PCR. They summarized that comparing results of the three different methods showed that HPV DNA was found only with nested PCR. The results presented imply that nested PCR is the most appropriate method for the detection of HPV DNA in FFPE samples. These results became the basis for us for strategy of detection of active HPV infection. This strategy was presented in paper of Janecka-Widła et al. [28].

In the present study, we have found P16 positivity in two HPV16 positive BC, whereas all HPV negative BC in PCR analysis were also negative for P16 expression. Expression of P16 is a known surrogate marker of HPV infection [46]. However, it should be noticed that overproduction of P16 can be caused not only by HPV infection, but also by oncogenes activation, DNA damage or accelerated cellular senescence [47]. In turn, genetic alteration of *P16* gene (deletion, methylation and point mutation), found in nearly 50% of malignancies, can inhibit synthesis of this protein [47]. Therefore, it can be expected that in case of a larger number of HPV16 positive BC divergences between PCR analysis and P16 immunoexpression could reveal themselves, as for example in the case in our analyses of head and neck [28] or rectal cancers [48].

## Conclusion

Presented by us results demonstrated very low percentage (0.5%) in breast tumors of patients from south-central Poland, what suggest that in this region HPV infection has no influence on development of breast tumors. Our study has some limitation related with the fact that it is case study without control group. However, case–control study is not suitable to study rare exposures, as in the case of the presented analysisy, in which the percentage of BC positivity is at the level of 0.5. It should be also noticed that in the light of contrary results concerning HPV prevalence in BC, future studies are needed to fully explain the association between this infection and development of breast cancer.

## Data Availability

The datasets generated during and/or analyzed during the current study are available from the corresponding author on reasonable request.

## References

[1] De Martel C, Georges D, Bray F, Ferlay J, Clifford GM (2020). Global burden of cancer attributable to infections in 2018: a worldwide incidence analysis. Lancet Glob Health.

[2] Bae M, Kim EH (2016). Human papillomavirus infection and risk of breast cancer: a meta-analysis of case-control studies. Infect Agents Cancer.

[3] Haghshenas MR, Mousavi T, Moosazadeh M, Afshari M (2016). Human papillomavirus and breast cancer in Iran: a meta-analysis. Iran J Basic Med Sci.

[4] Zhou Y, Li J, Ji Y, Ren M, Pang B, Chu M (2015). Inconclusive role of human papillomavirus infection in breast cancer. Infect Agent Cancer.

[5] Simões PW, Medeiros LR, Simões Pires PD, Edelweiss MI, Rosa DD, Silva FR (2012). Prevalence of human papillomavirus in breast cancer: a systematic review. Int J Gynecol Cancer.

[6] Li N, Bi X, Zhang Y, Zhao P, Zheng T, Dai M (2011). Human papillomavirus infection and sporadic breast carcinoma risk: a meta-analysis. Breast Cancer Res Treat.

[7] Islam MS, Chakraborty B, Panda CK (2020). Human papilloma virus (HPV) profiles in breast cancer: future management. Ann Transl Med.

[8] Khodabandehlou N, Mostafaei S, Etemadi A, Ghasemi A, Payandeh M, Hadifar S (2019). Human papilloma virus and breast cancer: the role of inflammation and viral expressed proteins. BMC Cancer.

[9] Zhang N, Ma ZP, Wang J, Bai HL, Li YX, Sun Q (2016). Human papillomavirus infection correlates with inflammatory Stat3 signaling activity and IL-17 expression in patients with breast cancer. Am J Transl Res.

[10] Ohba K, Ichiyama K, Yajima M, Gemma N, Nikaido M, Qingqing Wu Q (2014). In vivo and in vitro studies suggest a possible involvement of HPV infection in the early stage of breast carcinogenesis via APOBEC3B induction. PLoS ONE.

[11] Bønløkke S, Blaakær J, Steiniche T, Høgdall E, Jensen SG, Hammer A (2018). Evidence of no association between human papillomavirus and breast cancer. Front Oncol.

[12] Kouloura A, Nicolaidou E, Misitzis I, Panotopoulou E, Kassiani T, Smyrniotis V (2018). HPV infection and breast cancer. Results of a microarray approach. Breast.

[13] Bakhtiyrizadeh S, Hosseini SY, Yaghobi R, Safaei A, Sarvari J (2017). Almost complete lack of human cytomegalovirus and human papillomaviruses genome in benign and malignant breast lesions in Shiraz, Southwest of Iran. Asian Pac J Cancer Prev.

[14] Doosti M, Bakhshesh M, Zahir ST, Shayestehpour M, Karimi-Zarchi M (2016). Lack of evidence for a relationship between high risk human papillomaviruses and breast cancer in iranian patients. Asian Pac J Cancer Prev.

[15] Eslamifar A, Ramezani A, Azadmanesh K, Bidari-Zerehpoosh F, Banifazl M, Aghakhani A (2015). Assessment of the association between human papillomavirus infection and breast carcinoma. Iran J Pathol.

[16] Li J, Ding J, Zhai K (2015). Detection of human papillomavirus DNA in patients with breast tumor in China. PLoS ONE.

[17] Vernet-Tomas M, Mena M, Alemany L, Bravo I, De Sanjosé S, Nicolau P (2015). Human papillomavirus and breast cancer: no evidence of association in a Spanish set of cases. Anticancer Res.

[18] Herrera-Romano L, Fernández-Tamayo N, Gómez-Conde E, Reyes-Cardoso JM, Ortiz-Gutierrez F, Ceballos G (2012). Absence of human papillomavirus sequences in epithelial breast cancer in a Mexican female population. Med Oncol.

[19] De Cremoux P, Thioux M, Lebigot I, Sigal-Zafrani B, Salmon R, Sastre-Garau X, Institut Curie Breast Group (2008). No evidence of human papillomavirus DNA sequences in invasive breast carcinoma. Breast Cancer Res Treat.

[20] Lindel K, Forster A, Altermatt HJ, Greiner R, Gruber G (2007). Breast cancer and human papillomavirus (HPV) infection: no evidence of a viral etiology in a group of Swiss women. Breast.

[21] Wrede D, Luqmani YA, Coombes RC, Vousden KH (1992). Absence of HPV 16 and 18 DNA in breast cancer. Br J Cancer.

[22] de Villiers EM, Sandstrom RE, zur Hausen H (2005). Presence of papillomavirus sequences in condylomatous lesions of the mamillae and in invasive carcinoma of the breast. Breast Cancer Res.

[23] Widschwendter A, Brunhuber T, Wiedemair A, Mueller-Holzner E, Marth C (2004). Detection of human papillomavirus DNA in breast cancer of patients with cervical cancer history. J Clin Virol.

[24] Kołodziej-Andrejuk S, Patyra K, Macieląg P, Mandziuk S, Pachnia D, Mazurkiewicz M (2013). The presence of HPV DNA in breast cancer. J Prev Clin Clin Res.

[25] Delgado-García S, Martínez-Escoriza J-C, Alba A, Martín-Bayón T-A, Ballester-Galiana H, Peiró G (2017). Presence of human papillomavirus DNA in breast cancer: a Spanish case-control study. BMC Cancer.

[26] Goldhirsch A, Winer EP, Coates AS, Gelber RD, Piccart-Gebhart M, Thürlimann B, Senn HJ (2013). Panel members personalizing the treatment of women with early breast cancer: highlights of the St Gallen international expert consensus on the primary therapy of early breast cancer. Ann Oncol.

[27] Biesaga B, Janecka A, Mucha-Małecka A, Adamczyk A, Szostek S, Słonina D, Halaszka K, Przewoźnik M (2016). HPV16 detection by qPCR method in relation to quantity and quality of DNA extracted from archival formalin fixed and paraffin embedded head and neck cancer tissues by three commercially available kits. J Virol Methods.

[28] Janecka-Widła A, Mucha-Małecka A, Majchrzyk K, Halaszka K, Przewoźnik M, Słonina D, Biesaga B (2020). Active HPV infection and its influence on survival in head and neck squamous-cell cancer. J Cancer Res Clin Oncol.

[29] Biesaga B, Szostek S, Klimek M, Jakubowicz J, Wysocka J (2012). Comparison of the sensitivity and specificity of real-time PCR and in situ hybridization in HPV16 and 18 detection in archival cervical cancer specimens. Folia Histochem Cytobiol.

[30] Lewis JS, Chernock RD, Ma XJ, Flanagan JJ, Luo Y, Gao GX (2018). Partial p16 staining in oropharyngeal squamous cell carcinoma: extent and pattern correlate with human papillomavirus RNA status. Mod Pathol.

[31] Duò D, Ghimenti C, Migliora P, Pavanelli MC, Mastracci L, Angeli G (2008). Identification and characterization of human papillomavirus DNA sequences in Italian breast cancer patients by PCR and line probe assay reverse hybridization. Mol Med Rep.

[32] Ghaffaria H, Nafissib N, Hashemi-Bahremanic M, Alebouyehd MR, Tavakolia A, Javanmarda D (2018). Molecular prevalence of umanpapillomavirus infection among Iranian women with breast cancer. Breast Dis.

[33] Ngamkham J, Karalak A, Chaiwerawattana A, Sornprom A, Thanasutthichai S, Sukarayodhin S (2017). Prevalence of human papillomavirus infection in breast cancer cells from Thai women. Asian Pac J Cancer Prev.

[34] Piana AF, Sotgiu G, Muroni MR, Cossu-Rocca P, Castiglia P, De Miglio MR (2014). HPV infection and triple-negative breast cancers: an Italian case-control study. Virol J.

[35] Kroupis C, Markou A, Vourlidis N, Dionyssiou-Asteriou A, Lianidou ES (2006). Presence of high-risk human papillomavirus sequences in breast cancer tissues and association with histopathological characteristics. Clin Biochem.

[36] De Carolis S, Pellegrini A, Santini D, Ceccarelli C, De Leo A, Alessandrini F (2018). Liquid biopsy in the diagnosis of HPV DNA in breast lesions. Future Microbiol.

[37] Salman NA, Davies G, Majidy F, Shakir F, Akinrinade H, Perumal D (2017). Association of high risk human papillomavirus and breast cancer: a UK based study. Sci Rep.

[38] Cavalcante JR, Porto Pinheiro LG, Carvalhode Almeida PR, Pitombeira Ferreira MV, Cruz GA, Campelo TA (2018). Association of breast cancer with human papillomavirus (HPV) infection in Northeast Brazil: molecular evidence. Clinics.

[39] Islam S, Dasgupta H, Roychowdhury A, Bhattacharya R, Mukherjee N, Roy A (2017). Study of association and molecular analysis of human papillomavirus in breast cancer of Indian patients: clinical and prognostic implication. PLoS ONE.

[40] Wang Y-W, Zhang K, Zhao S, Lv Y, Zhu J, Liu H (2017). HPV status and its correlation with BCL2, p21, p53, Rb, and survivin expression in breast cancer in a Chinese population. Biomed Res Int.

[41] De Carolis S, Storci G, Ceccarelli C, Savini C, Gallucci J, Sansone P (2019). HPV DNA associates with breast cancer malignancy and it is transferred to breast cancer stromal cells by extracellular vesicles. Front Oncol.

[42] Corbex M, Bouzbid S, Traverse-Glehen A, Aouras H, McKay-Chopin S, Carreira C (2014). Prevalence of papillomaviruses, polyomaviruses, and herpesviruses in triple-negative and inflammatory breast tumors from Algeria compared with other types of breast cancer tumors. PLoS ONE.

[43] Habyarimana T, Attaleb M, Mazarati JB, Bakri Y, El Mzibri M (2018). Detection of human papillomavirus DNA in tumors from Rwandese breast cancer patients. Breast Cancer.

[44] Erhart SM, Rivero ER, Bazzo ML, Onofre AS (2016). Comparative evaluation of the GP5+/6+, MY09/11 and PGMY09/11 primer sets for HPV detection by PCR in oral squamous cell carcinomas. Exp Mol Pathol.

[45] Božić L, Jovanović T, Šmitran A, Janković M, Knežević A (2020). Comparison of HPV detection rate in formalin-fixed paraffin-embedded tissues of head and neck carcinoma using two DNA extraction kits and three amplification methods. Eur J Oral Sci.

[46] Serrano M (1997). The tumor suppressor protein p16INK4a. Exp Cell Res.

[47] Li J, Poi MJ, Tsai MD (2011). Regulatory mechanisms of tumor suppressor P16(INK4A) and their relevance to cancer. Biochemistry.

[48] Biesaga B, Janecka-Widła A, Kołodziej-Rzepa M, Słonina D, Darasz Z, Gasińska A (2019). The prevalence of HPV infection in rectal cancer—report from South-Central Poland (Cracow region). Pathol Res Pract.

